# “…or else I close my ears” How women with obesity want to be approached and treated regarding gestational weight management: A qualitative interview study

**DOI:** 10.1371/journal.pone.0222543

**Published:** 2019-09-19

**Authors:** Anne Christenson, Eva Johansson, Signy Reynisdottir, Jarl Torgerson, Erik Hemmingsson

**Affiliations:** 1 Obesity Center, Academic Specialist Center, Stockholm Health Services, Stockholm, Sweden; 2 Department of Medicine, Karolinska Institutet, Stockholm, Sweden; 3 Department of Public Health Sciences (IHCAR), Karolinska Institutet, Stockholm, Sweden; 4 Department of Molecular and Clinical Medicine, Institute of Medicine, Sahlgrenska Academy, University of Gothenburg, Göteborg, Sweden; 5 The Swedish School of Sports and Health Sciences, Åstrand Laboratory of Work Physiology, Stockholm, Sweden; University of Brighton, UNITED KINGDOM

## Abstract

**Introduction:**

The importance of helping pregnant women maintain a healthy lifestyle and prevent excessive gestational weight gain is well recognized, but pregnant women do not always perceive communication about body weight as respectful or helpful. Furthermore, fear of inducing shame or guilt can prohibit some midwives from talking about body weight, especially if the woman has obesity. We aimed to explore what women of reproductive age with obesity regard to be the most important and relevant aspects when discussing gestational weight management.

**Methods:**

Qualitative interview study using focus groups and individual semi-structured interviews with 17 women of reproductive age (19–39 y) with obesity. Thematic analysis was used to analyze the data.

**Results:**

We identified three themes: 1) Importance of obtaining vital medical information; 2) A wish to feel understood and treated with respect; 3) Midwives’ approach is crucial in sensitive key situations, which include bringing up the subject of body weight, weighing, providing weight-related information, coaching lifestyle modification, dealing with emotional reactions and ending a conversation.

**Conclusions:**

A majority of the interviewed women wished to receive information about risks about obesity and gestational weight gain, and recommendations on weight management. However, the risk of midwives offending someone by raising the topic may be increased if the pregnant woman believe that gestational weight gain is uncontrollable by the individual. Also, several situations during maternity care meetings can be stigmatizing and make women less receptive to advice or support. Women suggest that a good working alliance is likely to be achieved if midwives have knowledge about the causes of obesity, take interest in the patients’ background, have a non-judgmental approach and refrain from giving unsolicited advice.

## Introduction

Obesity is defined by the World Health Organization (WHO) as having a body mass index (BMI) ≥30 kg/m^2^ [1]. The proportion of women with obesity in the pregnant population is increasing across the world. In US, the prevalence has reached between 18–34% [2], and in European countries it varies between 7–25% [3]. In Sweden, the prevalence of obesity in pregnant women has reached between 10–22% depending on socio-demographic region [4,5].

Consequently, midwives frequently meet women whose body weight constitutes a risk for pregnancy-related complications, such as gestational diabetes, pre-eclampsia or stillbirth [6,7]. The majority of pregnant women with obesity underestimate their BMI, overestimate their recommended gestational weight gain and show poor knowledge about health risks associated with excess weight gain or maternal obesity [8,9]. The importance of raising awareness about recommendations, as well as helping pregnant women maintain a healthy lifestyle, and prevent excessive gestational weight gain is therefore a priority [10–12].

During the last ten years, there is an on-going process in Sweden to implement intervention programs to promote healthy lifestyle during pregnancy and to limit gestational weight gain in women with obesity [13,14]. Interventions generally include extra visits and rely on midwives to weigh pregnant women more often, disseminate relevant information, and support lifestyle modification. However, according to statements from parous women, communication about body weight in maternity health care is not always respectful or helpful, and the topic of gestational weight gain can instead be perceived as confusing, judgmental or even ignored [9,15–17].

Interview studies about how parous women with obesity have perceived their maternity care or interventions, suggest communication about body weight should be respectful and honest to create a satisfactory working alliance between the pregnant woman and her care-giver [18,19]. At the same time, due to awareness of weight stigma combined with empathy for the patient, midwives sometimes avoid talking about body weight so as to not inflict worries, shame or guilt in pregnant women [20,21]. Although this avoidant behavior is meant to save pregnant women from distress, it may also leave them unaware of risks and recommendations about healthy lifestyle and gestational weight gain [9], and thereby deprived of the ability to make informed decisions [22]. This indicates a need to further explore how pregnant women’s wishes and needs regarding gestational weight management may be met.

On this basis, we aimed to identify the opinions and wishes of women of reproductive age with obesity about what would be important aspects of good and successful gestational weight management, and their preferences for receiving advice about this matter from their pregnancy health care providers.

The research questions covered in the interviews concerned:

- How do women with obesity, who are planning to become pregnant, perceive the scenario that some midwives avoid talking about obesity-related risks and weight gain recommendations for fear of inducing worries or shame?
- How would women with obesity like to be approached and treated by maternity care staff in a future pregnancy regarding weighing and gestational weight management?

## Method

Qualitative methodology allows us to explore, interpret and describe participants’ experiences and perceptions of particular circumstances in specific contexts. We used focus groups and individual interviews including an introductory background scenario, to assess women’s views and opinions about gestational weight management. Focus groups were used to benefit from participants’ interactions to inspire and facilitate the discussion and thereby enhance data quality [23]. At the same time, social phobia is common in people with obesity [24], and some feel uncomfortable speaking in front of a group. Therefore, we used individual interviews as a complement to facilitate for participants who felt reluctant to speak in a group, or who could not attend the focus group dates, to choose the time and setting they felt the most comfortable with. Both focus groups and individual interviews are two well-established and effective ways to obtain data about a topic that may be of sensitive nature [23,25,26]. Also, the combination of two independent data collection methods has been suggested as a productive strategy to enrich data by gathering complementary views and thereby enhance the description of a phenomenon [27,28]. To analyze the data we used thematic analysis according to Braun and Clarke [29]. Interview method as well as the analysis are described in detail under the data collection and analysis section respectively.

### Study participants and recruitment

We recruited women of reproductive age with different levels of obesity (WHO obesity class I, II, III) from a publicly funded specialist clinic for obesity treatment in Stockholm, Sweden. Thus, we ensured exploring the views and attitudes of women who are likely to benefit from maintaining a healthy gestational weight gain, while also representing a population that some midwives perceive as difficult to approach due to the stigma of obesity [20]. Patients at the clinic are treatment seeking men and women, age ≥16 years with a BMI ≥ 30 kg/m^2^ and at least one comorbidity, where ordinary outpatient weight loss support have failed. The care at the specialist clinic include individually tailored support for lifestyle modification, e.g. regular meetings with a clinician, group lectures on diet and exercise and weight loss medication.

Women were considered eligible if they were between 18–39 years of age, fluent in Swedish, planning to have children, and had a BMI ≥ 30 kg/m^2^. For ethical reasons, pregnant women were excluded since the interviews include risk information, and we did not want to risk inducing worries in already pregnant women. Former experience from antenatal care were neither an inclusion nor exclusion criteria as we wanted women to speculate freely about how an ideal future contact with maternity care should be, without necessarily relating their wishes to earlier experiences. Flyers about the study were posted in the clinic and handed out by caregivers during regular visits. Written consent was provided from 17 women after receiving oral and written information about the study. Participants could choose what data collection method (focus group or individual interview) they preferred. Recruitment stopped when it was decided between three researchers that the retrieved data was rich, descriptions sufficiently in-depth and no more new data had emerged in the last three interviews, which indicated that further interviews would be redundant [23,30].

### Data collection

We performed three focus groups and six individual interviews with 17 participants in total from March to July 2018. The first group contained only two participants due to late cancellations, but discussions were vivid and rich. Groups number two and three consisted of four and five participants respectively.

All interviews took place at the specialist obesity outpatient clinic, where the participants were enrolled in standard obesity care. To describe population characteristics, data about height and weight were measured at the clinic, and data about degree of employment, marital status, parity and country of origin were gathered before each interview by letting each participant complete a short questionnaire.

Focus groups lasted (and were limited to) 90 min and were conducted by the first author, a female cognitive therapist/physiotherapist, with experience from leading group sessions and performing in-depth interviews, together with another clinician with experience from focus group interviews. Two focus group participants had been former patients to the interviewer while the others had no earlier established contact.

Since speculation about a possible future scenario may be difficult [31] and awareness about risks with excessive gestational weight gain in the general population is low [8,9], both focus groups and individual interviews commenced by a three-minute introduction, describing the risks associated with obesity during pregnancy, recommendations regarding gestational weight gain and the benefits of a healthy lifestyle. The use of introductory scenarios or vignettes can work as a facilitator when opening up a discussion and may be perceived as a non-threatening way of exploring sensitive topics [32,33]. The scenario, that some midwives may avoid talking about obesity risks and weight gain recommendations for fear of inducing guilt and shame in pregnant women, was also described. Women were then encouraged to express their thoughts and feelings about what they had heard.

The first author assumed an attitude of *emphatic neutrality* [23] while facilitating the discussion by asking participants to elaborate or to clarify their statements when needed e.g. *“When you say*, *‘midwives should be understanding’*, *what is it you would like them to understand*?*”* or *“How can you tell that they [the midwives] have understood you*?*”*. Questions were open ended and neutral to avoid biased answers [31]. A topic guide including the introduction scenario in writing and predetermined areas of interest (reactions to the scenario and wishes about midwives approach), was used by the interviewer to ensure that all participants received the same introduction scenario, and that areas of research interest were covered.

When necessary, in order to facilitate for women to articulate what approach they *preferred* (instead of automatically only describing what they *do not* want), participants were asked to imagine that the interviewer was a midwife student in need of concrete advice on how to act towards pregnant women with obesity. This role-play made it easier for women to formulate concrete wishes.

To ensure that everybody was given ample opportunity to express their thoughts and opinions, directed questions such as *“What are your thoughts on this*?*”* were used to engage less active participants in the discussion. Notes were made about body language, facial expressions and group interaction. At the end of the focus groups the facilitator made an oral summary to reflect back to the group, what factors, circumstances and stands had been mentioned, allowing the group to correct or add to the data.

Only the first author was present in the individual interviews that lasted between 29–48 min. The same introduction, questions and interview techniques were used as in the focus groups. Transcripts from individual interviews were returned to each participant for correction or comments. No corrections were demanded.

#### Ethical considerations

Providing information about recommendations and risks regarding weight gain in future pregnancies may have induced worries in some participants. However, we considered it to be less ethical to leave women unaware and we set aside time at the end of each interview for additional questions. In addition, participants could bring this up later in their regular meetings at the clinic.

The study was performed in line with the Helsinki declaration [34] and approved by The Regional Ethical Review Board, Stockholm (Dnr: 2017/2279-31/5).

### Analysis

We used inductive thematic analysis, following the six phases described by Braun and Clarke, to process the data [29]. Throughout the analysis process the first author noted down her perceptions and reflections on the material to be used as part of the self-reflexivity process. *Phase 1*. *Familiarizing yourself with your data*: Audio recordings from focus groups and individual interviews were transcribed verbatim by the first author and notes about non-verbal communication, such as group interaction, laughing or making illustrative gestures were added. The first author, who was already familiar with the data after interviewing and transcribing, read through all data several times, highlighting text passages, relevant for the research questions. For analyst triangulation [23], transcribed data from the first focus group were analyzed by two researchers independently and then compared to check that the same text passages were highlighted as relevant for the research questions. The few differences found were discussed and resolved, thus, making sure no important data were left out during the continued process. To ensure that early impressions or ideas had not progressively steered the analyst to exclude relevant text material this procedure was also done with the last analyzed individual interview. *Phase 2*. *Generating initial codes*: The first author labelled relevant passages with a code that captured either the semantic or the implicit meaning of the text while also noting reflections and initial ideas of themes. During this process, codes were sometimes changed, merged or renamed until the final codes were checked for approval from another researcher (EJ) experienced in qualitative methodology. *Phase 3*. *Searching for themes*. Four researchers (AC, EJ, EH, JT) proposed and discussed preliminary themes, their interconnections and how they related to the research questions. *Phase 4–5*. *Reviewing*, *defining and naming themes*: Preliminary tables with codes and thematic charts were developed and the process of re-visiting the original transcripts to confirm or re-name themes were done in frequent discussions during a period of six months, until final themes were given names that described the essential meaning of each theme.

Quotes were translated from Swedish to English by the first author and then checked by two English/Swedish-speaking peers.

## Results

### Participants’ characteristics

The study population varied in age, covered a wide range of BMI above 30, and a majority were Swedish born and in a relationship (Table 1).

**Table 1:** Characteristics of participants (n = 17).

Characteristics	Values
Age (y)	Mean 26.9, Median 26 (range 19–39)
Body weight (kg)	Mean 115.7, Median 115 (range 81.3–138.2)
BMI (kg/m^2^)	Mean 40.2, Median 39.4 (range 32.6–52.7)
In a relationship *	n = 14
Nulliparous	n = 14
Born outside Sweden	n = 3
Fully employed/ studying	n = 12

* As self-defined in questionnaire.

### Main findings

We formulated three themes regarding gestational weight management in maternity care. The first two themes: 1) *Importance of obtaining vital medical information*, and 2) *A wish to feel understood and treated with respect*, captures what women wish to obtain regarding medical and emotional aspects from a maternity care meeting. The third theme: 3) *Midwives’ approach is crucial in sensitive key situations*, concerns how women want to be approached and treated by their midwives in six specific sensitive situations: Bringing up the subject of body-weight, weighing, providing weight-related information, coaching lifestyle modification, dealing with emotional reactions, and ending a conversation.

### Importance of obtaining vital medical information (theme 1)

All women expressed concern about their baby’s health, and most women said that they wish to obtain vital information about how their obesity, gestational weight gain and lifestyle may affect the baby. Women were concerned about midwives who refrain from providing information due to fear of pregnant women’s emotional reactions.

“*My first thought is that it is completely mad not to bring it up*! *[…] I would like to know all risks there are*. *[…] It would be far more sensitive if you find out afterwards that something [bad] happened because of your weight*.*”* Individual interview no. 6

The influence of risk-awareness on health choices were discussed as women saw a risk of making unhealthy choices due to lack of knowledge. Knowing that your behaviors may have a direct effect on the health of your baby was considered an important motivator.

“*If you are aware of the risks I think you subconsciously think ‘Maybe I should be a bit more active and perhaps I shouldn’t have this unhealthy stuff and maybe eat a fruit instead of this bag of* crisps’” Individual interview no. 4

Opinions were divided, however, and three of the 17 women preferred that the topic about risks with obesity and excessive weight gain was not raised since it could make them worried. These were women who seemed to believe that the individual has very limited ability to control gestational weight.

*”But I don’t think you can control how much weight you gain during pregnancy*. *It depends on how large the baby gets and how much space it needs and then what cravings you have*. *[…] I mean*, *it [the baby] may get it [obesity] but I can’t do anything about it now*.*”* Individual interview no. 2

It was also mentioned that women with obesity have their own responsibility to be open for information.

*“We as patients have to be open to take in the information regarding our larger bodies and that we are having a child and yes*, *that it may increase the risks”* Focus group no. 2

### A wish to feel understood and treated with respect (theme 2)

Participants expressed the importance of being understood, listened to and taken seriously. They also described a good meeting with health care providers in general is when you are treated with respect, interest and a non-judgmental attitude. Importantly, women claimed that if these wishes were met by their midwife, they would be more open and honest, while the opposite could make them completely unapproachable to information or advice.

*“Or else it’s like ‘I’m not going to listen to you now’ [illustrates by covering her ears with her hands]*, *almost as if you push away what she said*. *I close my ears”* Focus group no. 1

“*I can get defensive if someone just tells me what to do*, *because then it feels like they don’t respect me or assume that I’m stupid or ignorant*.*”* Individual interview no. 1

Participants’ immediate response to the question of how they would like to be approached regarding the subject of healthy gestational weight gain, were to automatically share the countless number of previous bad encounters they had experienced in health care settings in general. Women reported that health professionals had made wrongful, weight biased assumptions about women’s food or exercise habits, given unsolicited advice, lectured about the dangers of being obese or commented on women’s weight in a harsh (tone of voice, threatening face expression, etc.) manner. When stories surfaced in focus groups about this stigmatizing approach, other focus group members were nodding in agreement, adding affirmative statements from own experiences.

*“I can’t count the number of times I’ve been told by caregivers to stop eating candy*, *[rolling her eyes] which I hardly ever do*, *[…] ‘but I assume you drink soda*?*’*, *which I don’t*!*”* Focus group no. 1

*”What I find hard when I go to the gynecologist or a midwife is that you get judged*. *‘Perhaps you should lose weight*!*’ when they could instead ask ‘have you tried losing weight*?*’ It feels like they are very quick at drawing the conclusion that you have chosen to be big”* Focus group no. 3

### Midwives’ approach is crucial in sensitive key situations (theme 3)

Women identified six specific sensitive situations that may arise in maternity care meetings. In each of them, midwives approach was considered crucial, and could either hinder or facilitate a positive patient-caregiver relationship and a good working alliance, depending on whether or not women’s wishes regarding medical aspects (theme 1), and treatment (theme 2) are met. Below, women’s wishes for midwives’ approach in each of the six situations are presented.

#### Bringing up the subject of body weight

Asking for permission before bringing up the topic of body weight was considered a sign of respect. Additionally, verbally acknowledging that it may be a sensitive matter indicates that the midwife is insightful which helps women to feel understood.

*“They can say ‘This may be a sensitive subject*, *but I have to bring it up with all pregnant women’ “*Individual interview no. 3

To avoid feeling judged and instead feel understood and listened to it was suggested that midwives use an open body language, e.g. uncrossed arms, face the patient, keep eye-contact, smile and show interest by asking the woman about her background including lifestyle, weight history and former knowledge about obesity.

*“Take me for example*. *I’m still overweight but I have lost a lot of weight so maybe they should ask about my background*: *-Tell me about your background*! *What does your ordinary day look like*? *How has your weight changed over the years*?*”* Focus group no. 1

#### Weighing

Although not all women felt uncomfortable to weigh themselves they all acknowledged that stepping on the scales is a sensitive issue to many women with obesity.

” A: What is it that makes it sensitive?

*I think it’s feeling ashamed about what you see on the scale*. *Even if people can see that you are overweight*, *with the weighing you kind of get it thrown in your face*.*”* Individual interview no. 4

To minimize stigma and shame, it was suggested that all women, regardless BMI, should be weighed at each visit. This would avoid feeling singled out as obese and also, it would be possible to detect excessive weight gain in women with BMI below 30.

*”Make it a routine to weigh everybody*. *[…] regardless of size*. *Because that would get rid of the judging part like ‘I think you look big so we’ll have to go and check on the scales […] Instead you can put everyone there [on the scales] and thereby also keep track of if normal weight women are gaining more than their recommendations*. Focus group no. 3

Any reluctance to weighing should be acknowledged and discussed in an empathic manner and psychological support may be offered.

”*If you don’t want to weigh yourself*, *there’s usually a reason for it*. *Perhaps it’s something associated [with weighing] that makes you really anxious […] Then perhaps it is time to say ‘well*, *we have a really good counselor here you could talk to’* Focus group no. 1

During weighing, women wanted to have the option not to see how much they weigh, and they wished that midwives should maintain a neutral facial expression regardless what numbers they see on the scale. Written information could proposedly serve several causes. If a woman refuses to weigh herself or decline talking about body weight, a folder could be handed out along with an offer to bring up the topic some other time. This was also suggested as an aid for midwives who feel uncomfortable to bring up weight.

*“…something you can hand out and say that “here is some information about risks associated with pregnancy etc*. *[…] Then midwives don’t have to talk about it if they find it hard*. Focus group no. 2

Using the right wording was frequently mentioned. Expressions like overweight, weight category, BMI and weight change were considered neutral or less negatively loaded than words like fat, obese or obesity.

*“And not use the word obesity or fat or obese*, *God no*! *Because those make you anxious […] ‘Weight change’*, *well…it doesn’t feel as loaded or negative as fat or obesity*. *‘Overweight’ is also a good expression*, *because I* ***am*** *overweight*. *So rather use that instead of ‘fat’ or ‘obese’*. *‘Fat’ feels more like a label or a value*.*”* Individual interview no 1

#### Providing weight-related information

Using scare tactics as a way to motivate lifestyle change was not considered a helpful approach and may make women discontinue treatment.

“*…because you cannot scare away a weight problem*. *It is not a good method*! *It doesn’t work*! *Unfortunately*! *Or else we would have been very thin*. *(laughs) […]I have met doctors who lectured me and made me go home and cry afterwards*. *It has never worked to frighten someone to lose weight*. *It only ads to your anxiety and worries and often has the opposite effect somehow*. Individual interview no. 1

*“Midwives must be allowed to say what they need to say but not in a way that…so that it doesn’t feel good to come back*. *Or else it’s like no*, *I won’t bother coming back because that midwife will only nag me”*. Focus group no. 1

#### Coaching lifestyle modification

The women wished that information about risks with obesity should always be accompanied by a treatment plan. If not, you may be left with anxiety and worries which in itself can induce overeating.

*"It would be great to receive the same kind of treatment as a patient with cancer*. *Because they have someone laying it out nicely*, *they get treatment plans*, *they receive help*. *We are often met by ‘Ok*, *this is a routine checkup and you can book another appointment later when you’d like to come back’*. *There is never a treatment plan whatsoever*.*"* Focus group no. 3

The women expressed a wish that midwives, as well as clinicians in general, should increase their knowledge and understanding about how difficult it is to lose weight if you have obesity.

“*I really think that society believes it’s quite easy to just lose weight if you are very large*, *as if there’s a quick-fix*, *something that is easily done*.*”* Focus group no. 2

*“I think one knows that it is unhealthy to be overweight*, *but you can quit smoking at once*, *but you can’t just quit being overweight*.*”* Focus group no. 3

Increased knowledge could also reduce the risk of making wrongful assumptions about women’s lifestyle.

*”…because there are people who have a very healthy lifestyle despite having a high BMI*, *and there are normal weight people who are malnourished or who have a very unhealthy diet but don’t gain weight*. *So*, *you shouldn’t assume that a person is in a certain way just because of her weight*.*”* Focus group no. 3

Women also requested that to better coach lifestyle, their midwives should be knowledgeable of current diet and exercise recommendations, focus on positive health messages and notice and encourage any positive behavior change women make.

*“Someone who can lift you up and see the positive instead of the negative…see the progress*. *[…] Because you are so good at being self-critical […] to talk about it in a way that is emphatic*, *understanding and where you focus on the positive*.*”* Individual interview no. 1

#### Dealing with emotional reactions

Midwives should not fear women’s emotional reactions but instead acknowledge them and ask about underlying causes. It was commonly expressed that raising the topic of obesity and difficulties in limiting weight gain can be sensitive and induce both fear, shame and sadness in pregnant women. Even so, comparison was made with how obviously inappropriate it would be for an oncologist to let fear of a patients’ emotional response prevent them from telling someone they have cancer and are in need of treatment.

*“Because sometimes it is as if they’re like ‘Oh no*, *she’s starting to cry’*. *Because it is sensitive*, *but midwives shouldn’t be afraid of feelings*. *They mustn’t let themselves be scared by emotions*.*”* Individual interview no. 1

*“…you still want to know*. *Or else it would be as if an oncologist would be avoidant and think that ‘She does have cancer but it’s just a little*, *so I don’t bother saying it*. *[…] That would be really stupid*. *[…] Just because I may get sad or offended you should not keep information that is important from me*. *Then it’s better to become upset at first and then talk about it*, *as in ‘I notice this makes you sad’ or ‘I may have offended you*, *and that may be*, *but this is an important subject’”* Individual interview no. 6

Women saw pregnancy as a time when difficult psychological issues may surface. However, emotional moments where, however, considered golden opportunities to assess if the woman need more time to talk, professional help from a psychologist, dietician or referral to a weight loss clinic.

*“Difficult and sensitive matters will surface and I would like midwives to have the time and space to address them*. *[…] If it is something more serious the midwife should be confident enough to [admit] that ‘I may not be able to handle this*. *And instead refer [the woman] to someone else*, *a counselor for example*.*”* Focus group no. 2

*“… it took many years before I even dared to go to the doctor and say that ‘I am sad and I need help and I feel ugly and fat’*. *[…] So I can see it as an opportunity [at the midwifes’]*. *[…] I for example was referred here [to the obesity clinic] or if you are sad because you were bullied then maybe you need to see a psychologist*.*”* Individual interview no. 6

#### Ending a conversation

When wrapping up a meeting midwives could ask about how women perceived the conversation. If the rapport between the pregnant woman and the midwife is unsatisfactory women wished it would be easy to swap midwife.

*“I think it would be good if*, *by the end of the meeting*, *you were asked about how you found the conversation*. *[…] You should have some sort of evaluation*, *which doesn’t have to be verbal*. *It can be written or digital*. *Something like ‘What did you think of this visit*?*’”* Focus group no. 2

## Discussion

### Main findings

This interview study indicates that many women want to receive information about how their weight, lifestyle and gestational weight gain may affect the health of their baby. However, several situations during maternity care meetings can be stigmatizing and make the woman less receptive to advice or support. It was therefore suggested that midwives become more knowledgeable about obesity, approach the topic of body weight by showing an interest in women’s background, and refrain from making wrongful assumptions resulting in unsolicited advice. Hereafter we discuss salient points in our findings to illustrate how these relate to earlier research, and how our findings may affect implications for clinical practice.

#### Sensitive situations as opportunities to create alliance

Our participants had previous experience from care-givers who gave advice without any knowledge about women’s former weight loss attempts or background. This behavior may be perceived as offensive and make women feel misunderstood or underestimated and thus counterproductive [19]. These findings concur with many studies who report that pregnant women with obesity feel judged or stigmatized [15,35–37], and confirm that a non-judgmental support from a caregiver who is well familiar with the topic of obesity would be desirable and supportive [18,38].

Since attitudes towards people with obesity are influenced by whether weight change is considered controllable by the individual or not [39,40], increased knowledge about the causes of obesity may lessen the risk of stigmatization from maternity care staff. This study adds the suggestion that women’s own beliefs about individual ability to control weight may influence both their interest in receiving information and increase the risk of becoming offended by midwives raising the topic of body weight. Since the etiology of obesity is a mix of genes and environment [41] we therefore agree with earlier studies who urge that the complexity of weight change is made known to health care professionals and also to the general public to reduce weight stigma and facilitate communication about gestational weight gain [35].

Regarding terminology, our findings add to previous studies showing that words like fat, obese or obesity are not neutral to people with obesity but are heavily loaded with negative emotions, and stereotypes, and therefore more neutral words like BMI or weight category are preferred [42,43]. For this reason, although the term obesity has a strict and useful medical definition, it may still be beneficial to use a less stigmatizing term when communicating with patients.

#### Weighing during pregnancy

In our, as well as in other studies, some women expressed reluctance towards being weighed [44], while other women who had been weighed during pregnancy were positive to the experience [44,45]. These varied opinions demonstrates a need to approach the weighing situation with care. Following the weight trajectory more frequently in women of all BMI-categories, and not just women with obesity, could possibly lessen the stigma and avoid women with obesity to feel singled out. It would also enable midwives to discover excessive gestational weight in normal weight women, who are otherwise rarely target for interventions, but nevertheless at risk for pregnancy related complications such as preeclampsia, gestational diabetes, fetal macrosomia, cesarean delivery [46,47] and postpartum weight retention [48]. Furthermore, the weighing situation provides an opportunity to detect situation bound anxiety and possibly reveal eating disorders which are otherwise hard to discover [49].

To further consider the sensitivity around body weight, interventions who focus on the nutritional quality of food, exercise or psychological wellbeing, and measure other outcomes than weight needs further exploration [50–52]. Especially since there still is a lack of consensus about optimal gestational weight gain in women with obesity class II and III [53,54] and some interventions do not achieve the intended outcomes, despite successful limitation of weight gain [55,56].

#### Communication tools

Many of the women’s suggestions on how to improve communication were in accordance with the patient-centered communication style taught in Motivational Interviewing (MI), e.g. using open questions, providing a non-judgmental atmosphere, exploring people’s own reasons for change and develop a plan for action [57]. Women’s expectations for medical staff to be weight biased may make women particularly sensitive to midwives’ behaviors which further emphasizes the importance of communication skills. In weight interventions, the use of MI seems to improve outcomes [58,59]. Furthermore, teaching MI to maternity care staff has been shown to make midwives more aware of their communication style, increase their confidence in bringing up the subject of obesity, improve their competence in talking to pregnant women with obesity and reduce work-related stress [60–62]. Many Swedish midwives have various levels of training in MI. Studies show, however, that, among caregivers who claim to be using MI, the true use of MI tools varies substantially between [63,64], and also over time within individuals [65].

#### Need for additional professional support

With higher prevalence of obesity in the pregnant population an increasing number of risk pregnancies and greater need for additional professional support will follow. Some interventions include access to a dietician which has been appreciated by women [66]. Our patients suggest there may be a hidden prevalence of psychological issues in many women with obesity, as do midwives in earlier findings [67]. However, while women with obesity wish their regular maternity care should include time to address psychological issues and to support mental well-being, some midwives instead avoid asking about sensitive issues in fear of inducing painful emotional responses in women, or due to lack of time or professional support [20].

Midwives’ ability, or inability, to cope with women’s emotional reactions or specific obesity-related questions was frequently mentioned both in focus groups and in individual interviews. It is therefore plausible that access to a team of other professionals such as psychologists/counselors as well as dieticians and physiotherapists may positively affect women’s health and also improve midwives’ work situation by reducing the stress of feeling inadequate or time constraints [20,22,67].

#### Strengths and limitations

One may argue that interviewing women about a hypothetical future situation provide less useful data than asking about actual lived experiences. However, while earlier interview studies have assessed women’s opinions to evaluate intervention programs, we sought to explore the opinions and wishes of a population who were planning to become pregnant, without necessarily relating or limiting their suggestions to earlier maternity care experiences. In this case, using the introduction vignette followed by imaginative future scenarios may therefore have put less boundaries on their fantasy and creative thinking when they described what an ideal communication and midwife-patient relation should be like [32].

Recruiting women from one obesity clinic may be a limiting factor. However, the wide range of obesity levels (class I, II, III) and the variation of age and socio-demographic background in our population, increases the possibility to apply the findings to other women with obesity in similar medical settings [68]. The fact that all participants had experienced the patient-centered communication style used at the obesity clinic may have influenced their thoughts on the matter. This can however also be seen as a strength as it adds to their lived experiences of different communication styles. Performing the study at an obesity friendly and, to participants, familiar setting with an experienced therapist as interviewer may also have allowed the women to feel particularly comfortable and safe, enabling them to open up and talk honestly about negative experiences as well as hopes and wishes about future gestational health management.

#### Conclusions

This study indicates that many women of reproductive age with obesity want information about risks and recommendations about obesity and gestational weight gain. This study further suggests that women with obesity often expect midwives to be weight biased and the risk of midwives offending someone by raising the topic is increased if the pregnant woman believes that gestational weight gain is uncontrollable by the individual. Several specific situations during maternity care meetings can be stigmatizing and make women less receptive to advice or support. A good working alliance is likely to be achieved if midwives have knowledge about the causes of obesity, take interest in the patients’ background, use a non-judgmental approach and refrain from giving unsolicited advice.

## Supporting information

S1 File Interview guide in Swedish (original). (DOCX)

S2 File Interview guide in English. (DOCX)
