# Supplementary material for: “…or else I close my ears” How women with obesity want to be approached and treated regarding gestational weight management: A qualitative interview study
Source: PLoS One. 2019 Sep 19;14(9):e0222543. doi: 10.1371/journal.pone.0222543 (PMC6752788; doi:10.1371/journal.pone.0222543)
Supplement: S1 File — (DOCX) [file pone.0222543.s001.docx]

ORIGINAL SWEDISH VERSION

Intervjuguide Fokusgrupp/ intervju Fokusgrupp nr:

Antal deltagare:

Bisittare:

Intervju nr:

1. Presentera mig, studien samt bisittarens funktion (för FG)
2. Repetera studieinformationen och att deltagarna är källor till kunskap men att man kan välja att inte svara eller säga något
3. Önskan om konfidentialitet mellan deltagarna i gruppen! Det vi säger här idag i gruppen, stannar i gruppen.
4. Samla in
   1. Samtycke
   2. Bakgrundsinformation
5. Ramar för gruppen
   1. Tala en i taget, (underlättar avlyssningen)
   2. Sluttid
   3. Mailar inte utskrift av deltagares uttalanden vid fokusgrupp (utan bara vid enskilda intervjuer) Summerar vid slutet av gruppen och ber deltagarna kolla av om jag uppfattat rätt.
   4. Om någon har ytterligare frågor eller funderingar efter gruppen angående fetma och graviditet eller studien går det bra att kontakta mig eller ta upp med sin handledare.

STARTA INSPELNING!

1. *Kort presentationsrunda*. Säg några ord om dig själv (för ljudkoll och identifikation av röster)!

*Presentera scenariot LÄS:*

*Eftersom vikten ökar i befolkningen är det också fler gravida kvinnor som har ett BMI >30. När man jämför gravida kvinnor som har BMI>30 med de som har <30 ser man att komplikationer och graviditetsbesvär är vanligare bland de som har fetma. Det är t.ex. vanligare med högt blodtryck, diabetes, stora barn eller svåra förlossningar. Det är också vanligare att barnet drabbas av hjärtfel, tidig spädbarnsdöd eller själv får fetma eller diabetes senare i livet.*

*Men man har också sett att riskerna kan påverkas (minskas) av hur mycket man går upp i vikt under graviditeten och vilka levnadsvanor man har. Bäst tycks vara om man har ett lägre BMI redan innan man blir gravid, men det finns ändå mycket man kan göra för hälsan om man har ett högt BMI när man blir gravid.*

*För gravida kvinnor med BMI >30 är rekommendationen idag att man ska gå upp mellan 5-9 kg.*

*Om man delar upp den grupp som har BMI>30 och jämför de som går upp enligt rekommendationerna med de som går upp över rekommendationerna ser man att det är fler som har en frisk och okomplicerad graviditet i gruppen som lyckas att inte gå upp mer än rekommenderat.*

*Idag känner de allra flesta barnmorskor till det här. Samtidigt, (och här kommer en av de saker som jag vill att ni ska reflektera kring och ge era synpunkter och tankar på), samtidigt som vi har kunskap om vilka risker som finns vid fetma och vad man kan göra för att påverka dem (t.ex. leva hälsosamt och begränsa viktuppgång), så visar studier att en del barnmorskor drar sig för att väga kvinnor och ta upp och prata om vikten för att det kan vara känsligt och man inte vill kränka eller oroa kvinnor eller få dem att känna skuld och skam över sin vikt eller viktuppgång. En del barnmorskor väljer därför ibland att tona ned riskerna, anpassa viktuppgångsråden till kvinnan de har framför sig, eller undviker att prata om vikten helt och hållet. En del barnmorskor avstår från att väga kvinnan om de tror att kvinnan inte vill det.*

*Å ena sidan gör man detta, individanpassar informationen, för att inte oroa eller kränka kvinnan. Å andra sidan blir följden att kvinnor kan få olika information och en del gravida kvinnor med BMI >30 får otydlig information eller inget samtal alls om hur vikt och viktutveckling kan påverka graviditeten och barnet.*

**Del 1 Reaktioner på scenariot**

- Nu skulle jag vilja öppna den här diskussionen med att få höra: Vad tänker och tycker ni spontant om den här informationen och scenariot som jag beskrev?

**Del 2 Önskan om framtida bemötande**

- Om du blir gravid framöver, hur skulle du vilja att barnmorskorna handskades med det här ämnet (vägning, viktrekommendationer och samtal om levnadsvanor)? Du får önska och säga helt fritt.

Möjliga extra fördjupningsämnen om de inte kommer upp automatiskt:

- Vad ska de säga och hur?
- Vad ska de göra om du/kvinnan inte vill (väga sig, prata om vikten)?
- Är det något mer du/ni vill nämna som kan vara viktigt för barnmorskor att veta ?
