# Supplementary material for: “…or else I close my ears” How women with obesity want to be approached and treated regarding gestational weight management: A qualitative interview study
Source: PLoS One. 2019 Sep 19;14(9):e0222543. doi: 10.1371/journal.pone.0222543 (PMC6752788; doi:10.1371/journal.pone.0222543)
Supplement: S2 File — (DOCX) [file pone.0222543.s002.docx]

TRANSLATED ENGLISH VERSION

Interview guide Focus group / interview Focus group no.:

Number of participants:

Assistant:

Interview #:

1. Presentation of me, the study and the function of the assistant (for focus groups)

2. Repeat the study information and that the participants are sources of knowledge but that you can choose not to answer or say anything

3. The desire for confidentiality between the participants in the group! What we are saying here today in the group, stays in the group.

4. Collect

a. Written consent

b. Background Information

5. General information for the group

a. Speak one at a time, (to facilitate listening to the recording)

b. Time frames

c. Only email the participant's statements at individual interviews. Summarizes at the end of the group and asks the participants to check if I understood correctly.

d. If someone has any further questions or concerns about the group regarding obesity and pregnancy or the study, please contact me or consult with their supervisor.

START RECORDING!

6. Short presentation round. Say a few words about yourself (for audio check and voice identification)!

Presenting the scenario READ:

*As weight increases in the population, there are also more pregnant women who have a BMI> 30. When comparing pregnant women who have BMI> 30 with those who have <30, it has been shown that complications and pregnancy-related problems are more common among women with obesity. It is for example more common with high blood pressure, diabetes, large babies or complications in childbirth. It is also more common for the child to suffer from heart failure, early infant death or develop obesity or diabetes later in life.*

*But it has also been shown that the risks may be influenced (decreased) by how much weight you gain during pregnancy and what lifestyle habits you have. The best scenario appears to be if you have a lower BMI even before you get pregnant, but there is still much you can do for your health if you have a high BMI when you become pregnant.*

*For pregnant women with BMI> 30, the recommendation today is to gain between 5-9 kg.*

*If you divide the group that has BMI> 30 and compare those who gain according to the recommendations with those who gain more than the recommendations, it has been shown that there are more healthy and uncomplicated pregnancies in the group that manage to below the recommended upper limit.*

*Today, the vast majority of midwives know this. At the same time, (and here is one of the things that I want you to reflect upon and give your views and thoughts on), while we have knowledge of existing risks with obesity and what you can do to influence those risks (e.g. living healthy and limit weight gain), studies show that some midwives are reluctant to weigh women with obesity and talk about body weight because it can be sensitive, and sometimes midwives fear it may offend or worry women or make them feel guilt and shame about their weight or weight gain. Some midwives therefore choose to downplay the risks, adjust the weight gain advice to the woman they have in front of them, or avoid talking about the weight altogether. Some midwives refrain from weighing the woman if they think the woman does not want it.*

*On one hand midwives do this, tailor the information for the individual, as to not to worry or offend the woman. On the other hand, it means that women receive different information. And some pregnant women with BMI> 30 get unclear information or no conversation at all about how weight and weight development can affect pregnancy and the child.*

**Part 1 Reactions to the scenario**

- Now I would like to open this discussion by hearing what you have to say: What are you spontaneous thoughts and reactions to the information and scenario that I described?

**Part 2 Wishes for future treatment**

- If you get pregnant in the future, how would you like the midwives to handle this subject (weighing, weight recommendations and conversation about lifestyle habits)? Please feel completely free to say or wish anything you come to think of regarding this matter.

Possible extra topics, if they do not automatically come up:

- What should midwives say and in what way?
- What should they do if you/ a woman does not want to get weighed or talk about body weight?
- Is there anything more you would like to mention that may be important for midwives to know ?
